# Supplementary material for: Elevated blood remnant cholesterol and triglycerides are causally related to the risks of cardiometabolic multimorbidity
Source: Nat Commun. 2024 Mar 19;15:2451. doi: 10.1038/s41467-024-46686-x (PMC10951224; doi:10.1038/s41467-024-46686-x)
Supplement: Supplementary file 1 — Supplementary Information [file 41467_2024_46686_MOESM1_ESM.pdf]

Supplementary Fig. 1. Disease transition models. Panels: (a) pooling all three cardiometabolic disease together; (b) only pooling ischemic heart disease and stroke together. Abbreviations: IHD, ischemic heart disease; T2D, type 2 diabetes.

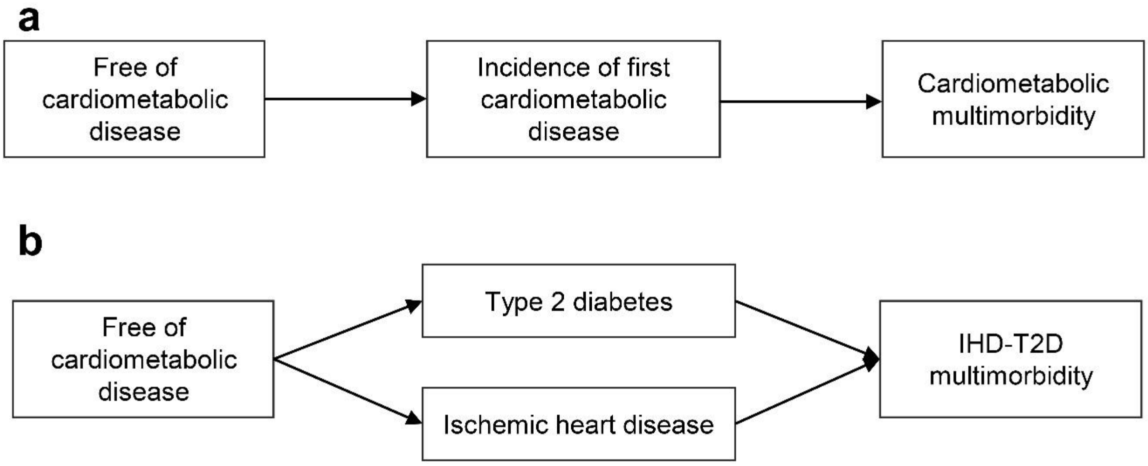

Supplementary Fig. 2. Genetic associations between remnant cholesterol, triglycerides, and the three cardiometabolic diseases via one-sample Mendelian randomization among the UK Biobank participants (n = 376,712 for remnant cholesterol and n = 411,930 for triglycerides). Causal odds ratios were calculated adjusting for age and sex. Data are presented as odd ratios with 95% confidence interval. The dot refers to the odd ratio while the solid line refers to the 95% confidence interval. The dash line refers to odd ratio value of one as the reference. Abbreviations: CI, confidence interval; OR, odds ratio. Statistical significance is set at two-sided *P* value < 0.05.

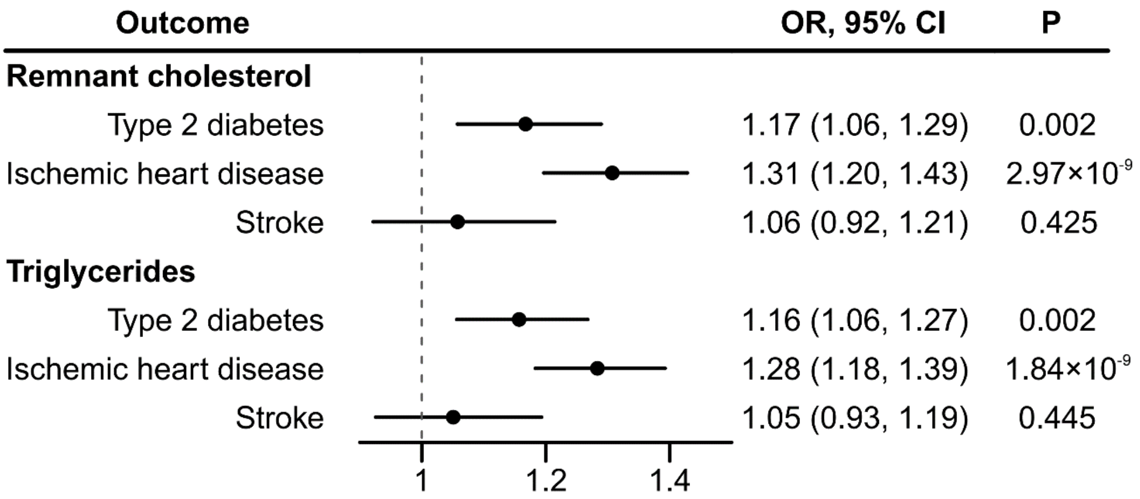

Supplementary Fig. 3. Participants flow chart. Panels: (a) participants included in the observational analysis; (b) participants included in the Mendelian randomization analysis.

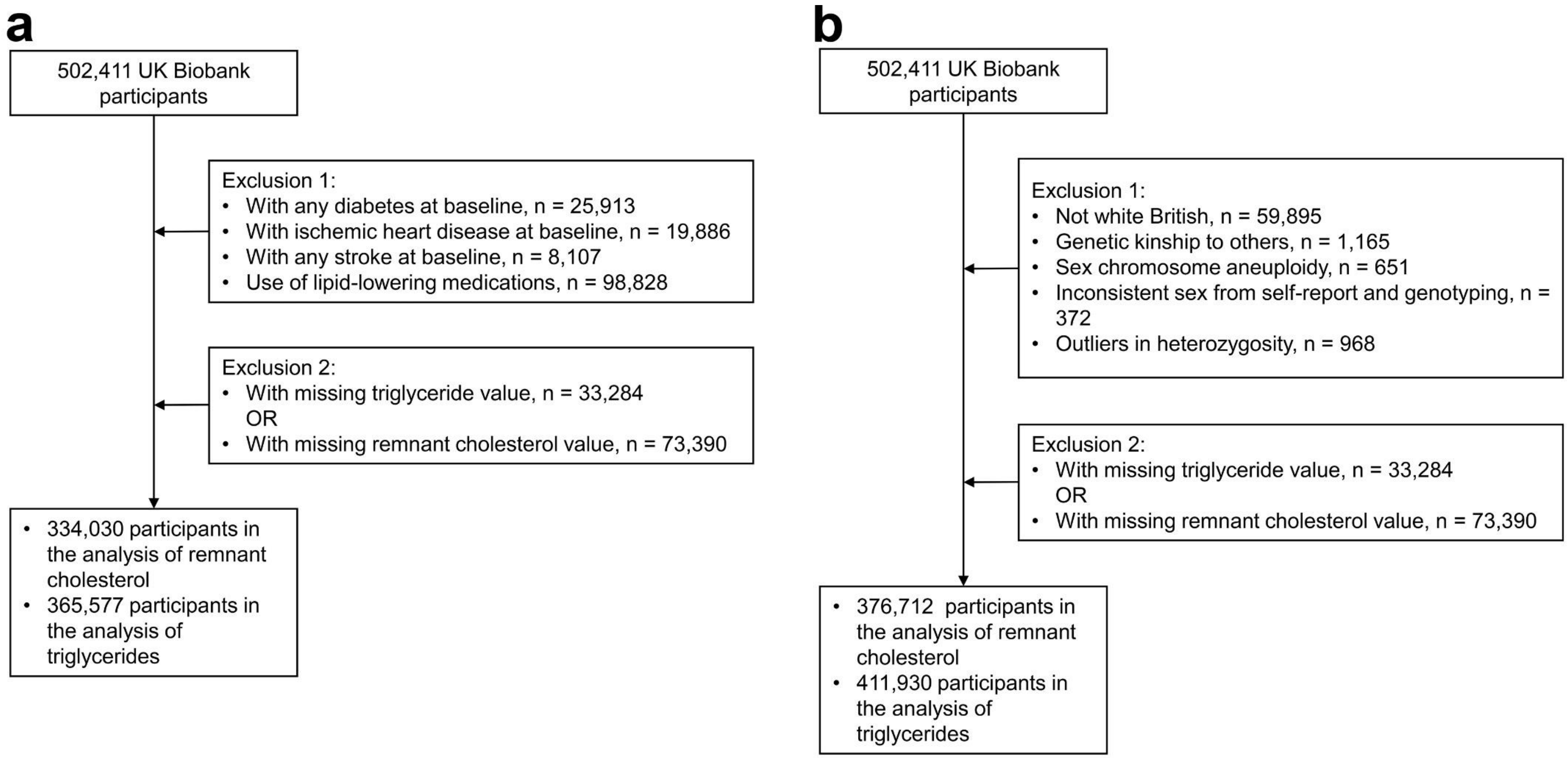

STROBE-MR checklist of recommended items to address in reports of Mendelian randomization studies

Item No.	Section	Checklist item	Page No.	Relevant text from manuscript
1	**TITLE and ABSTRACT**	Indicate Mendelian randomization (MR) as the study's design in the title and/or the abstract if that is a main purpose of the study	2	We also evaluated the causality and shapes of these associations via Mendelian randomization using 13 biologically relevant SNPs as the genetic instruments
	**INTRODUCTION**			
2	**Background**	Explain the scientific background and rationale for the reported study. What is the exposure? Is a potential causal relationship between exposure and outcome plausible? Justify why MR is a helpful method to address the study question	3	It is noteworthy that diet, like fat and cholesterol intake, and lifestyle factors might substantially influence triglyceride-rich lipoprotein metabolism.[19-21] The complex interplay between lipid species might also confound the observational relationships between triglyceride-rich lipoproteins and cardiometabolic diseases.[22]
3	**Objectives**	State specific objectives clearly, including pre-specified causal hypotheses (if any). State that MR is a method that, under specific assumptions, intends to estimate causal effects	4	In this study, we utilized multistate modeling, two-stage least squares regression-based Mendelian randomization, doubly-ranked stratification-based non-linear Mendelian randomization to investigate the associations between remnant cholesterol, triglycerides and the risks of cardiometabolic multimorbidity in the UK Biobank.
	**METHODS**			
4	**Study design and data sources**	Present key elements of the study design early in the article. Consider including a table listing sources of data for all phases of the study. For each data source contributing to the analysis, describe the following:		
	a)	Setting: Describe the study design and the underlying population, if possible. Describe the setting, locations, and relevant dates, including periods of recruitment, exposure, follow-up, and data collection, when available.	14	The UK Biobank is a prospective study of over 500,000 UK adults aged between 37 and 73 years that were recruited between 2006 and 2010.[36]
	b)	Participants: Give the eligibility criteria, and the sources and methods of selection of participants. Report the sample size, and whether any power or sample size calculations were carried out prior to the main analysis	15	In the Mendelian randomization analysis, participants were excluded if they were not

				white British (n = 59,895), did not pass the quality control for genotyping (n = 3636), were prescribed lipid-lowering medications (n = 98,828), or had missing values for remnant cholesterol (n = 73,390) or triglycerides (n = 33,284). Therefore, 301,565 and 330,031 eligible participants were included in the Mendelian randomization analysis of remnant cholesterol and triglycerides, respectively (Supplementary Fig. 2b).
	c)	Describe measurement, quality control and selection of genetic variants	16	A total of 13 biologically relevant SNPs were included as genetic instruments for remnant cholesterol and triglycerides (Supplementary Table 7).
	d)	For each exposure, outcome, and other relevant variables, describe methods of assessment and diagnostic criteria for diseases	16-17	The prevalent and newly on-set cardiometabolic diseases, namely type 2 diabetes, ischemic heart disease, and stroke, were identified via linkage to primary care records, hospital inpatient records, and death registry records and outcomes were coded using the International Classification of Disease, 10th revision codes: type 2 diabetes, E11; ischemic heart disease, I21-I25; stroke, I60-I64. We censored participants at the end of follow-up, at the date of the first occurrence of any cardiometabolic disease or development of multimorbidity, and at the date of loss to follow-up, whichever was first. Cardiometabolic multimorbidity was defined as the occurrence of at least two cardiometabolic diseases, like the co-existence of ischemic heart disease and type 2 diabetes (IHD-T2D multimorbidity) and

				the co-existence of stroke and ischemic heart disease (IHD-stroke multimorbidity).
		e) Provide details of ethics committee approval and participant informed consent, if relevant	14	Ethical approval for the UK Biobank was obtained from the North West Multi-center Research Ethics Committee (11/NW/0382).
5	**Assumptions**	Explicitly state the three core IV assumptions for the main analysis (relevance, independence and exclusion restriction) as well assumptions for any additional or sensitivity analysis		
6	**Statistical methods: main analysis**	Describe statistical methods and statistics used		
		a) Describe how quantitative variables were handled in the analyses (i.e., scale, units, model)		
		b) Describe how genetic variants were handled in the analyses and, if applicable, how their weights were selected	16	We calculated weighted GRSs as aggregated genetic instrumentals for blood remnant cholesterol and triglycerides. The weighted GRS was calculated by summing the products of the number of lipid-raising alleles and the effect size of each SNP on blood remnant cholesterol and triglycerides (Supplementary Table 7).
		c) Describe the MR estimator (e.g. two-stage least squares, Wald ratio) and related statistics. Detail the included covariates and, in case of two-sample MR, whether the same covariate set was used for adjustment in the two samples	19-20	We next conducted doubly-ranked stratification-based non-linear Mendelian randomization analysis to explore the shapes of the causal associations between remnant cholesterol, triglycerides, and cardiometabolic multimorbidity. Eligible participants (Supplementary Fig. 2) were first ranked according to their GRS values to form pre-stratum, and then ranked within each pre-stratum according to their blood lipid values (serum remnant cholesterol/triglycerides) to form ten strata. Unlike traditional residual stratification, doubly-ranked stratification could relax the strong parametric assumptions of linearity and homogeneity between the instrument and the exposure to form the strata.[50] Within each stratum, we calculated the localized average causal effect (LACE)

					estimates for the associations between remnant cholesterol and triglycerides with cardiometabolic multimorbidity. The LACE estimates were combined to produce a non-linear association using the fractional polynomial methods.[51] The besting fitting fractional polynomial was chosen based on the likelihood function. Potential non-linearity was tested by comparing the non-linear fractional polynomial model compared with a linear model.[51] Non-linear Mendelian randomization was performed using the “SUMnlmr” package in R.[51]
		d)	Explain how missing data were addressed	NA	
		e)	If applicable, indicate how multiple testing was addressed	NA	
7	**Assessment of assumptions**		Describe any methods or prior knowledge used to assess the assumptions or justify their validity	19-20	
8	**Sensitivity analyses and additional analyses**		Describe any sensitivity analyses or additional analyses performed (e.g. comparison of effect estimates from different approaches, independent replication, bias analytic techniques, validation of instruments, simulations)	20	
9	**Software and pre-registration**				
		a)	Name statistical software and package(s), including version and settings used	21	Statistical analysis was performed in Stata/MP version 17.0 and R version 4.2.2 with significance set at P < 0.05.
		b)	State whether the study protocol and details were pre-registered (as well as when and where)	NA	
	**RESULTS**				
10	**Descriptive data**				
		a)	Report the numbers of individuals at each stage of included studies and reasons for exclusion. Consider use of a flow diagram	14	Please see Supplementary material.

		b)	Report summary statistics for phenotypic exposure(s), outcome(s), and other relevant variables (e.g. means, SDs, proportions)	28	
		c)	If the data sources include meta-analyses of previous studies, provide the assessments of heterogeneity across these studies	NA	
		d)	For two-sample MR:<br>i. Provide justification of the similarity of the genetic variant-exposure associations between the exposure and outcome samples<br>ii. Provide information on the number of individuals who overlap between the exposure and outcome studies	NA	
11	**Main results**				
		a)	Report the associations between genetic variant and exposure, and between genetic variant and outcome, preferably on an interpretable scale	7-9	Please see the Results in the manuscript
		b)	Report MR estimates of the relationship between exposure and outcome, and the measures of uncertainty from the MR analysis, on an interpretable scale, such as odds ratio or relative risk per SD difference	7-9	Please see the Results in the manuscript
		c)	If relevant, consider translating estimates of relative risk into absolute risk for a meaningful time period	NA	
		d)	Consider plots to visualize results (e.g. forest plot, scatterplot of associations between genetic variants and outcome versus between genetic variants and exposure)	31-32	Please see the Results in the manuscript
12	**Assessment of assumptions**				
		a)	Report the assessment of the validity of the assumptions		Not applicable in this study.
		b)	Report any additional statistics (e.g., assessments of heterogeneity across genetic variants, such as I^2, Q statistic or E-value)		Not applicable in this study.
13	**Sensitivity analyses and additional analyses**				
		a)	Report any sensitivity analyses to assess the robustness of the main results to violations of the assumptions		Not applicable in this study.
		b)	Report results from other sensitivity analyses or additional analyses		
		c)	Report any assessment of direction of causal relationship (e.g., bidirectional MR)		Not applicable in this study.

		d) When relevant, report and compare with estimates from non-MR analyses		
		e) Consider additional plots to visualize results (e.g., leave-one-out analyses)		
	**DISCUSSION**			
14	**Key results**	Summarize key results with reference to study objectives	9	Please see the Discussion in the manuscript
15	**Limitations**	Discuss limitations of the study, taking into account the validity of the IV assumptions, other sources of potential bias, and imprecision. Discuss both direction and magnitude of any potential bias and any efforts to address them	13	Please see the Discussion in the manuscript
16	**Interpretation**			
		a) Meaning: Give a cautious overall interpretation of results in the context of their limitations and in comparison with other studies	9-11	Please see the Discussion in the manuscript
		b) Mechanism: Discuss underlying biological mechanisms that could drive a potential causal relationship between the investigated exposure and the outcome, and whether the gene-environment equivalence assumption is reasonable. Use causal language carefully, clarifying that IV estimates may provide causal effects only under certain assumptions	12	Please see the Discussion in the manuscript
		c) Clinical relevance: Discuss whether the results have clinical or public policy relevance, and to what extent they inform effect sizes of possible interventions	12-13	Please see the Discussion in the manuscript
17	**Generalizability**	Discuss the generalizability of the study results (a) to other populations, (b) across other exposure periods/timings, and (c) across other levels of exposure	12-13	Please see the Discussion in the manuscript
	**OTHER INFORMATION**			
18	**Funding**	Describe sources of funding and the role of funders in the present study and, if applicable, sources of funding for the databases and original study or studies on which the present study is based	24	This work was supported by the National Natural Science Foundation of China (grant numbers: 82173499 and 82204026).
19	**Data and data sharing**	Provide the data used to perform all analyses or report where and how the data can be accessed, and reference these sources in the article. Provide the statistical code needed to reproduce the results in the article, or report whether the code is publicly accessible and if so, where	21	The original data that support the findings of this study are available from UK Biobank (https://www.ukbiobank.ac.uk/), but restrictions

				apply to the availability of these data, which were used under license for the current study, and so are not publicly available. Source data are provided with this paper.<br>Doubly-ranked stratification-based Mendelian randomization was conducted using the SUMnlmr R package: https://github.com/amymariemason/SUMnlmr.<br>Multistate model was developed using the stmerlin stata command: https://github.com/RedDoorAnalytics/stmerlin.<br>Forest plots were developed using the R package: https://cran.r-project.org/web/packages/forestploter.<br>Analytical codes have been deposited at https://github.com/yiminzhao/trl-and-multimorbidity.
20	**Conflicts of Interest**	All authors should declare all potential conflicts of interest	25	**Competing interests**<br>None.

This checklist is copyrighted by the Equator Network under the Creative Commons Attribution 3.0 Unported (CC BY 3.0) license.
