# Supplementary material for: Elevated blood remnant cholesterol and triglycerides are causally related to the risks of cardiometabolic multimorbidity
Source: Nat Commun. 2024 Mar 19;15:2451. doi: 10.1038/s41467-024-46686-x (PMC10951224; doi:10.1038/s41467-024-46686-x)
Supplement: Supplementary file 3 — Description of Additional Supplementary Files [file 41467_2024_46686_MOESM3_ESM.pdf]

Description of Additional Supplementary Files

Supplementary Data 1. Baseline characteristics of participants stratified by the quintiles of genetic risk scores for blood remnant cholesterol and triglycerides.

Supplementary Data 2. Serum concentrations of remnant cholesterol and triglycerides stratified by fasting time.

Supplementary Data 3. Observational associations between remnant cholesterol, triglycerides, and the three cardiometabolic diseases.

Supplementary Data 4. Subgroup analysis for the observational associations between remnant cholesterol, triglycerides, and IHD-T2D multimorbidity.

Supplementary Data 5. Predictive performance for IHD-T2D multimorbidity in models with and without remnant cholesterol or triglycerides.

Supplementary Data 6. Predictive performance for cardiometabolic multimorbidity in models with and without remnant cholesterol or triglycerides.

Supplementary Data 7. Genetic instruments for remnant cholesterol and triglycerides
