# Supplementary material for: Elevated blood remnant cholesterol and triglycerides are causally related to the risks of cardiometabolic multimorbidity
Source: Nat Commun. 2024 Mar 19;15:2451. doi: 10.1038/s41467-024-46686-x (PMC10951224; doi:10.1038/s41467-024-46686-x)
Supplement: Supplementary file 11 — Reporting Summary [file 41467_2024_46686_MOESM11_ESM.pdf]

nature portfolio

Corresponding author(s): Tao Huang

Last updated by author(s): Feb 23, 2024

Reporting Summary

Nature Portfolio wishes to improve the reproducibility of the work that we publish. This form provides structure for consistency and transparency in reporting. For further information on Nature Portfolio policies, see our Editorial Policies and the Editorial Policy Checklist.

Statistics

For all statistical analyses, confirm that the following items are present in the figure legend, table legend, main text, or Methods section.

n/a	Confirmed	
☐	☒	The exact sample size (*n*) for each experimental group/condition, given as a discrete number and unit of measurement
☐	☒	A statement on whether measurements were taken from distinct samples or whether the same sample was measured repeatedly
☐	☒	The statistical test(s) used AND whether they are one- or two-sided<br>*Only common tests should be described solely by name; describe more complex techniques in the Methods section.*
☐	☒	A description of all covariates tested
☐	☒	A description of any assumptions or corrections, such as tests of normality and adjustment for multiple comparisons
☐	☒	A full description of the statistical parameters including central tendency (e.g. means) or other basic estimates (e.g. regression coefficient) AND variation (e.g. standard deviation) or associated estimates of uncertainty (e.g. confidence intervals)
☐	☒	For null hypothesis testing, the test statistic (e.g. *F*, *t*, *r*) with confidence intervals, effect sizes, degrees of freedom and *P* value noted<br>*Give P values as exact values whenever suitable.*
☐	☒	For Bayesian analysis, information on the choice of priors and Markov chain Monte Carlo settings
☐	☒	For hierarchical and complex designs, identification of the appropriate level for tests and full reporting of outcomes
☐	☒	Estimates of effect sizes (e.g. Cohen's *d*, Pearson's *r*), indicating how they were calculated

Our web collection on statistics for biologists contains articles on many of the points above.

Software and code

Policy information about availability of computer code

Data collection	Stata/MP version 17.0 and R version 4.2.2
Data analysis	Multistate model was developed using the stmerlin stata command: https://github.com/RedDoorAnalytics/stmerlin. Forest plots were developed using the R package: https://cran.r-project.org/web/packages/forestploter. Analytical codes have been deposited at Zenodo (https://doi.org/10.5281/zenodo.10560862).

For manuscripts utilizing custom algorithms or software that are central to the research but not yet described in published literature, software must be made available to editors and reviewers. We strongly encourage code deposition in a community repository (e.g. GitHub). See the Nature Portfolio guidelines for submitting code & software for further information.

Data

Policy information about availability of data

All manuscripts must include a data availability statement. This statement should provide the following information, where applicable:

- Accession codes, unique identifiers, or web links for publicly available datasets
- A description of any restrictions on data availability
- For clinical datasets or third party data, please ensure that the statement adheres to our policy

This research was conducted using the UK Biobank Resource (https://www.ukbiobank.ac.uk/) under Application ID 44430. Data from UK Biobank is accessible to

eligible researchers via applying to www.ukbiobank.ac.uk. Data supporting the findings of this study are available in the article and its Supplementary information. Source data are provided with this paper.

Research involving human participants, their data, or biological material

Policy information about studies with human participants or human data. See also policy information about sex, gender (identity/presentation), and sexual orientation and race, ethnicity and racism.

Reporting on sex and gender	We reported the percentage of the sex of the participants as follows: Therefore, 334,030 (men% = 42.5%) and 365,577 (men % = 42.1%) eligible participants were included in the observational analysis of remnant cholesterol and triglycerides, respectively (Supplementary Fig. 2a). Therefore, 376,712 (men% = 46.2%) and 411,930 (men% = 45.9%) eligible participants were included in the Mendelian randomization analysis of remnant cholesterol and triglycerides, respectively (Supplementary Fig. 2b).
Reporting on race, ethnicity, or other socially relevant groupings	In the Mendelian randomization analysis, participants were excluded if they were not white British (n = 59,895).
Population characteristics	The UK Biobank is a prospective study of over 500,000 UK adults aged between 37 and 73 years that were recruited between 2006 and 2010.
Recruitment	The UK Biobank is a prospective study of over 500,000 UK adults aged between 37 and 73 years that were recruited between 2006 and 2010.
Ethics oversight	The UK Biobank has obtained ethical approval from the North West Multi-centre Research Ethics Committee as a Research Tissue Bank approval (Approval Number: 11/NW/0382, https://www.ukbiobank.ac.uk/learn-more-about-uk-biobank/about-us/ethics). Besides, we also got approval for performing data analysis from Peking University Third Hospital Medical Science Research Ethics Committee (Approval Number: S2024080).

Note that full information on the approval of the study protocol must also be provided in the manuscript.

Field-specific reporting

Please select the one below that is the best fit for your research. If you are not sure, read the appropriate sections before making your selection.

☒ Life sciences ☐ Behavioural & social sciences ☐ Ecological, evolutionary & environmental sciences

For a reference copy of the document with all sections, see nature.com/documents/nr-reporting-summary-flat.pdf

Life sciences study design

All studies must disclose on these points even when the disclosure is negative.

Sample size	We reported sample size in the Methods and Result section
Data exclusions	We reported participant inclusion and exclusion in the Result section
Replication	This is a prospective analysis of UK Biobank. We did not perform replication in other population.
Randomization	This study is not a clinical trial involving randomization.
Blinding	This study is not a clinical trial involving blinding.

Reporting for specific materials, systems and methods

We require information from authors about some types of materials, experimental systems and methods used in many studies. Here, indicate whether each material, system or method listed is relevant to your study. If you are not sure if a list item applies to your research, read the appropriate section before selecting a response.

Materials & experimental systems

n/a	Involved in the study
☒	☐ Antibodies
☒	☐ Eukaryotic cell lines
☒	☐ Palaeontology and archaeology
☒	☐ Animals and other organisms
☒	☐ Clinical data
☒	☐ Dual use research of concern
☒	☐ Plants

Methods

n/a	Involved in the study
☒	☐ ChIP-seq
☒	☐ Flow cytometry
☒	☐ MRI-based neuroimaging

Plants

Seed stocks	Not applicable to this study.
Novel plant genotypes	Not applicable to this study.
Authentication	Not applicable to this study.
